# Supplementary material for: Discovering the Bioactive and Antibacterial Potential of Essential Oils from Aromatic Plants of Northeastern Peru
Source: Molecules. 2025 Oct 30;30(21):4236. doi: 10.3390/molecules30214236 (PMC12610044; doi:10.3390/molecules30214236)
Supplement: Supplementary file 1 [file molecules-30-04236-s001.zip › Table S1.pdf]

Table S1. Main (≥5%) and minor compounds detected in the extracted essential oils

Essential oils	Location (UTM coordinates: zone, x, y, elevation)	Extraction yield (%) (Time to drop of the first drop of essential oil (min))		Main Compounds	Minor Compounds
		Dry season (Jul-Sept 2023)	Rainy season (Feb-Mar 2024)		
Zanthoxylum fagara (espino)	El Huito Sector, Jaen. (17, 742073, 9370455, 865m)	1.50 (42)	0.93 (35)	caryophyllene (40.00%), hedicariol (10.86%), (-)-cis-β-elemene (9.08%), 1,5,9,9,9-tetramethyl-1,4,7-cycloundecatriene (5.86%)	guaiol (4.48%), δ-amorphene (3.77%), copaene (3.26%), α-epi-7-epi-5-eudesmol (3.10%), α-muurolene (2. 17%), (1S,2E,6E,10R)-3,7,11,11-tetramethylbicyclo[8.1.0]undeca-2,6-diene (2.05%), γ-eudesmol (1.84%), bulnesol (1. 73%), (1aR,4aR,7S,7aR,7bR)-1,1,7-Trimethyl-4-methylenedecahydro-1H-cyclopropa[e]azulen-7-ol (1.63%), γ-muurolene (1.20%), cis-β-copaene (1. 07%), α-cadinol (1.00%), δ-elemene (0.99%), γ-elemene (0.96%), Ledol (0.87%), (-)-α-cubebene (0.74%), γ-gurjunene (0. 72%), (3S,3aR,3bR,4S,7R,7aR)-4-Isopropyl-3,7-dimethyloctahydro-1H-cyclopenta[1,3]cyclopropa[1,2]benzen-3-ol (0.49%), nerolidol (0. 42%), β-selinene (0.41%), 5-azulenemethanol, 1,2,3,4,5α,6,7,8-octahydro-α,α,3β,8β-tetramethyl-, acetate (0. 23%), (1S,3aS,4S,5S,7aR,8R)-5-isopropyl-1,7a-dimethyloctahydro-1H-1,4-methanoinden-8-ol (0.20%), τ-cadinol (0.18%), trans-Calamenene (0. 17%), β-borbonene (0.16%), (-)-α-gurjunene (0.09%), D-Limonene (0.08%), α-fellandrene (0.07%), sabine (0.07%), p-cymene (0.05%).
Piper amalago (matico)	Road to La Virginia, Jaen (17, 730878, 9366686, 2135m)	0.60 (34)	0.83 (38)	(+)-δ-cadinene (13.63%), 1,4,7,-cyclondecatriene, 1,5,9,9-tetramethyl-, Z,Z,Z- (10.50%), β-pinene (9.96%), .γ.-muurolene (8.26 %), apiol (7.21%). cyclofenchene (6.02%)	eucalyptol (4.50%), β-copaene (4.21%), 1H-cycloprop[e]azulen-7-ol, decahydro-1,1,7-trimethyl-4-methylene-, [1ar-(1a.α.,4a.α.,7.β.,7a.β.,7a.β.,7 b.α.)] (3.73%), caryophyllene (3.23%), copaene (3.16%), (3R,3aR,3bR,4S,7R,7aR)-4-Isopropyl-3,7-dimethyloctahydro-1H-cyclopenta[1,3]cyclopropa[1,2]benzen-3-ol (2. 34%), L-α-terpineol (1.73%), (-)-α-cubebene (1.73%), Naphthalene, 1,2,3,5,6,7,7,8,8a-octahydro-1,8a-dimethyl-7-(1-methylethenyl)-, [1R-(1.α.,7 .β.,8 a.α.)]- (1.70%), β-copaene (1.68%), humulene epoxide II (1.47%), α-epi-7-epi-5-eudesmol (1.40%), (+)-trans-calamenene (1. 31%), 3,8-dimethyl-5-α-hydroxy-δ^9-octa-hydroazulene acetate (0.93%), (-)-α-gurjunene (0.84%), α-muurolene (0.83%), β-elemene (0.78%), α-cadinol (0.76%), β-calachorene (0. 71%), D-Limonene (0.71%), 4a(2H)-Naphthalenol, 1,3,4,5,6,8a-hexahydro-4,7-dimethyl-1-(1-methylethyl)-, (1S,4S,4aS,8aR)- (0.69%), (+)-ylangene (0. 55%), naphthalene, 1,2,3,4,4,4a,7-hexahydro-1,6-dimethyl-4-(1-

					methylethyl)- (0.53%), 1-epi-Bicyclesquifellandrene (0.52%), τ-cadinol (0.41%), (-)-terpinen-4-ol (0.40%), α-amorphene (0. 37%), β-myrcene (0.29%), p-cymene (0.28%), (-)-β-borbonene (0.24%), α-terpinyl acetate (0.22%), cadin-3,5-diene (0.19%), (+)-calarene (0.18%), (+)-sabinol (0. 17%), 7R,8R-8-Hydroxy-4-isopropylidene-7-methylbicyclo[5.3.1]undec-1-ene (0.16%), (+)-borneol (0.16%), sabinene (0.15%), γ-terpinene (0.15%), myristicin (0. 15%), δ-elemene (0.12%), (-)-Myrtenol (0.10%), α-terpinolene (0.08%), α-tujene (0.08%), α-terpinene (0.08%), camphene (0.08%), peruviol (0.07%), teaspirane (0. 06%), citral (0.06%), α-thujenal (0.05%), neral (0.04%), myrtenyl acetate (0.03%), (1S,3S,5S)-1-isopropyl-4-methylenebicyclo[3.1.0]hexan-3-yl acetate (0.02%).
Piper aduncum L. (matico)	Road to Loma Santa, Jaen (17, 741031, 9370222, 1151m)	0.52 (35)	0.66 (35)	myristicin (38.26 %), caryophyllene (11.10 %), 1H-cycloprop[e]azulen-7-ol, decahydro-1,1,7-trimethyl-4-methylene-,[1ar-(1a.α.,4a.α.,7.β.,7 a.β,7b.α.)]- (10.04 %), isoaromadendrene epoxide (8.40 %), (3R,3AR,3BR,4S,7R,7AR)-4-Isopropyl-3,7-dimethyloctahydro-1H-cyclopenta[1,3]cyclopropan[1,2]benzen-3-OL (6.63 %), pentadecane (5.86 %).	β-copaene (3.12%), apiol (3.08%), 1,4,7,-cyclondecatriene, 1,5,9,9-tetramethyl-, Z,Z,Z- (2.47%), (-)-cis-β-elemene (2. 30%), (+)-δ-cadinene (2.01%), α-elemene (1.96%), τ-cadinol (1.68%), humulene epoxide II (1.62%), copaene (0.74%), trans-nerolidol (0.73%).
Piper glabribaccum (matico macho)	La Virginia, Jaen (17, 731081, 9365499, 2102m)	0.33 (30)	0.56 (40)	(-)-α-cubebene (14.30 %), trans-nerolidol (11.46 %), (-)-cis-β-elemene (9.63 %), guaiol (8.19 %), copaene (5.25 %), (-)-α-gurjunene (5.22 %)	bicyclo[5.2.0]nonane, 2-methylen-4,8,8-trimethyl-4-vinyl- (4.93%), α-selinene (4.51%), cadin1(10),4-diene (4.30%), 1H-cycloprop[e]azulen-7-ol, decahydro-1,1,7-trimethyl-4-methylene-, [1ar-(1a.α.,4a.α.,7 .β,7a.β,7b.α.)]- (3.62%), 1,4,7,-cyclondecatriene, 1,5,9,9-tetramethyl-, Z,Z,Z- (3.54%), alloaromadendrene (2. 85%), (3R,3AR,3BR,4S,7R,7AR)-4-isopropil-3,7-dimetiloctahidro-1H-ciclopenta[1,3]ciclopropa[1,2]bencen-3-OL (2.17%), 4a,5-dimetil-3-(prop-1-en-2-il)-1,2,3,4,4a,5,6,7-octahidronaftaleno (2.12%), cadaleno (2. 08%), eudesm-7(11)-en-4-ol (1.67%), γ-selinene (1.60%), trans-calamenene (1.46%), τ-cadinol (1.35%), humulene epoxide II (1.14%), β-calacorene (1. 10%), (-)-globulol (1.06%), 1,2,9,10-tetradehydroaristolane (0.97%), Z-β-guaiene (0.85%), γ-gurjunene (0.80%), 7R,8R-8-Hydroxy-4-isopropylidene-7-methylbicyclo[5. 3.1]undec-1-ene (0.59%), (1S,2E,6E,10R)-3,7,11,11-tetramethylbicyclo[8.1.0]undeca-2,6-diene (0.51%), 6-methyl-5-hepten-2-one (0. 49%), 1H-cicloprop[e]azulen-4-ol,decahidro-1,1,4,7-tetrametil-,[1ar-(1aα,4β,4aβ,7α,7aβ,7bα)]- (0.39%), α-corocaleno (0.33%), 6-isopropenil-4,8a-dimetil-1,2,3,5,6,7,8,8a-octahidro-naftalen-2-ol (0. 22%), phytol (0.19%),

					cubenene (0.18%), isoaromadendrene epoxide (0.18%), α-amorphene (0.16%), γ-elemene (0.13%), disoxylonene (0.11%), citral (0. 09%), isoledene (0.09%), 1-octadecanol (0.09%), hexahydrofarnesyl acetone (0.06%), 6-Methyl-3,5-heptadien-2-one (0.01%), benzaldehyde (0.01%)
Esembeckia cornuta (botiyo)	Shanango Sector, Jaen (17, 748776, 9377978, 710m)	0.13 (54)	0.17 (40)	caryophyllene (16.79 %), δ-amorphene (14.58 %), (-)-cis-β-elemene (6.58 %), peruviol (6.23 %), (1aR,4aR,7S,7aR,7bR)-1,1,7-trimethyl-4-methylenedecahydro-1H-cyclopropa[e]azulen-7-ol (6.13 %), humulane-1,6-dien-3-ol (5.50 %)	copaene (3.94%), phytol (3.54%), 1,5,9,9,9-tetramethyl-1,4,7-cycloundecatriene (2.95%), (-)-7-Epi- α-selinene (2.43%), caryophyllene oxide (2.40%), β-borbonene (2.36%), apiol (2. 19%), α-epi-7-epi-5-eudesmol (2.03%), 2-propenoic acid, 3-[4-[(3-methyl-1-butenyl)oxy]phenyl]-, methyl ester (1.95%), hexadeca-2,6,10,14-tetraen-1-ol, 3,7,11,16-tetramethyl- (1. 80%), cis-β-copaene (1.21%), β-copaene (1.18%), liguloxide (1.16%), α-cadinol (1.13%), α-amorphene (1.08%), tau-cadinol (1.06%), 2-tert-butylquinoline (1.01%), hedicariol (0. 96%), cadin-3,5-diene (0.81%), gamma-muurolene (0.73%), 1,4-diisopropylbenzene (0.73%), 1H-cycloprop[e]azulen-4-ol, decahydro-1,1,4,7-tetramethyl-, [1ar (1aα,4β,4aβ,7α,7aβ,7bα)]- (0. 72%), (.+/-.)-cadinene (0.69%), 5-Azulenemethanol, 1,2,3,4,4,5α,6,7,8-octahydro-α,α,3β,3β,8β-tetramethyl-, acetate (0. 67%), (3S,3aR,3bR,4S,7R,7aR)-4-Isopropyl-3,7-dimethyloctahydro-1H-cyclopenta[1,3]cyclopropa[1,2]benzen-3-ol (0.66%), oxo-tremorine (0.64%), γ-elemene (0.57%), palmitic acid (0.56%), β-calacorene (0. 45%), dehydrofukinone (0.35%), γ-gurjunene (0.33%), trans-calamenene (0.31%), (-)-α-gurjunene (0.29%), myrcene (0.19%), homosalate (0.19%), L-α-terpineol (0. 17%), linalool (0.14%), methyl palmitate (0.13%), citral (0.12%), eucalyptol (0.11%), α-fellandrene (0.07%), neral (0.07%), β-tujene (0.06%), p-cymene (0.06%)
Magnolia manguillo (manguillo)	San Luis del retiro, Jaen (17, 727758, 9373433, 2328m)	0.13 (46)	0.07 (40)	(1aR,4aR,7S,7aR,7bR)-1,1,7-Trimethyl-4-methylenedecahydro-1H-	α-cadinol (4.94%), 1,5,9,9-tetramethyl-1,4,7-cycloundecatriene (4.81%), δ-amorphene (4.44%), isospatulenol (3. 89%), (1R,7S,E)-

				cyclopropa[e]azulen-7-ol (15.00 %), caryophyllene (11.59 %), cis-β-copaene (8.59 %), trans-nerolidol (6.46 %), copaene (5.89 %)	7-isopropil-4,10-dimetilenciclodec-5-enol (3.03%), 7R,8R-8-Hidroxi-4-isopropiliden-7-metilbiciclo[5.3. 1]undec-1-ene (2.76%), τ-cadinol (2.38%), γ-muurolene (2.31%), humulene epoxide II (2. 19%), 6-isopropenyl-4,8a-dimethyl-1,2,3,5,6,7,8,8,8a-octahydro-naphthalen-2-ol (1.40%), 1,5-epoxy-4(14)-salvialene (1.34%), (-) spatulenol (1. 28%), α-cubebene (1.21%), farnesol isomer a (1.17%), phytol (0.97%), 3-(2-isopropyl-5-methylphenyl)-2-methylpropionic acid (0.94%), ((4aS,8S,8aR)-8-isopropyl-5-methyl-3,4,4a,7,8,8,8a-hexahydronaphthalen-2-yl)methanol (0. 93%), calamenene (0.89%), salvial-4(14)-en-1-one (0.85%), ylangenol (0.82%), α-muurolene (0.80%), β-borbonene (0. 73%), (3S,3aR,3bR,4S,7R,7aR)-4-Isopropil-3,7-dimetiloctahidro-1H-ciclopenta[1,3]ciclopropa[1,2]bencen-3-ol (0. 72%), 2-isopropenyl-4a,8-dimethyl-1,2,3,4,4,4a,5,6,7-octahydronaphthalene (0.69%), caryophyllene oxide (0.65%), (-)-guaia-6,9-diene (0.63%), 4-terpineol, (+/-)- (0.54%), cis-muurola-4(15),5-diene (0.51%), apiol (0. 49%), β-terpinene (0.41%), palmitic acid (0.40%), ((8R,8aS)-8-Isopropyl-5-methyl-3,4,6,7,8,8,8a-hexahydronaphthalen-2-yl)methanol (0.38%), β-calacorene (0.38%), seline-3,7(11)-diene (0.31%), D-limonene (0. 25%), bicyclo[3.1.0]hexan-3-ol, 4-methylene-1-(1-methylethyl)-, [1S-(1α,3α,5α)]-(9CI) (0.23%), cedrene-V6 (0.21%), myrtenal (0.19%), β-tujene (0.19%), p-cymene (0.16), (-)-α-gurjunene (0. 15%), 1,4-dimethyl-4-vinylcyclohexene (0.15%), linalool (0.13%), cis-3-hexen-1-ol (0.12%), myrtenol, (-)- (0.11%), citral (0.09%), α-terpineol (0.07%), perylene (0.07%), α-tujene (0.06%), myrcene (0.05%), neral (0.05%)
Magnolia jaenensis (negrillo)	La Virginia, Jaen (17, 730913, 9366757, 2130m)	0.04 (46)	0.10 (35)	(-)-cis-β-Elemene (23.59 %), 2-Methyl-1-pentene (16.70 %), caryophyllene (15.26 %), apiol (14.08 %) and δ-amorphene (10.15 %)	1,5,9,9-tetramethyl-1,4,7-cycloundecatriene (2.64%), γ-selinene (2.43%), γ-muurolene (2.24%), β-selinene (1.88%), trans-nerolidol (1.21%), α-muurolene (1. 09%), caryophyllene oxide (1.07%), τ-cadinol (0.85%), (1aR,4aR,7S,7aR,7bR)-1,1,7-Trimethyl-4-methylenedecahydro-1H-cyclopropa[e]azulen-7-ol (0. 82%), β-copaene (0.74%), neointermedeol (0.61%), bicyclosesquiphellandrene (0.58%), citral (0.46%), α-cadinol (0.29%), myristicin (0. 29%), neral (0.28%), selin-6-en-4α-ol (0.27%), β-pinene (0.25%), trans- α-bisabolene (0.24%), oxo-tremorine (0. 21%), (3S,3aR,3bR,4S,7R,7aR)-4-Isopropyl-3,7-dimethyloctahydro-1H-cyclopenta[1,3]cyclopropa[1,2]benzen-3-ol (0.19%), β-calacorene (0. 17%), cadin-3,5-diene (0.16%), eucalyptol (0.14%), 6-isopropenyl-4,8a-dimethyl-1,2,3,5,6,7,8,8a-octahydro-naphthalen-2-ol (0.14%), humulene epoxide II (0. 13%), linalool (0.12%), L-α-terpineol (0.11%), (+)-γ-elemene

					(0.11%), cyclofenchene (0.11%), pentadecanal (0.08%), thymol (0. 08%), cubenene (0.07%), (-)-7-epi- α-selinene (0.06%), cis-β-copaene (0.05%), isobornyl acetate (0.03%), trans-2-decenal (0.02%)
Tessaria integrifolia (pájaro bobo)	Amoju River, Jaen (17, 739952, 9368225, 851m)	0.07 (40)	0.30 (40)	2-naphthalenomethanol, decahydro-α,α,4a-trimethyl-8-methylene-, [2R-(2 α,4aα,8aβ)]- (18.28 %), phenol, 2,4-bis(1,1-dimethylethyl)-6-methyl- (10.01 %), caryophyllene (9.00 %), 3,8-dimethyl-5-α-hydroxy-δ^9-octa-hydroazulene acetate (8. 74 %), 6,7-dimethyl-1,2,3,5,8,8,8a-hexahydronaphthalene (8.63 %), dihydroagarofuran (8.08 %), dehydrofukinone (6.82 %) and 4,4a,5,6,7,8-Hexahydro-4a,8-dimethylnaphthalen-2(3H)-one (6.71 %)	agarospirole (2.14%), δ-amorphene (2.11%), caryophyllene oxide (2.09%), (3R,3AR,3BR,4S,7R,7AR)-4-Isopropyl-3,7-dimethyloctahydro-1H-cyclopenta[1,3]cyclopropa[1,2]benzen-3-ol (2.03%), (-)-cis-β-elemene (1. 81%), (1aR,4aR,7S,7aR,7bR)-1,1,7-trimethyl-4-methylenedecahydro-1H-cyclopropa[e]azulen-7-ol (1.67%), copaene (1.53%), 1,5,9,9-tetramethyl-1,4,7-cycloundecatriene (1. 22%), apiol (1.01%), oplopenone (0.97%), (+)-sabinol (0.91%), naphthalene, 1,2,3,4-tetrahydro-1,1,2,4,4,7-hexamethyl (0. 75%), 4a(2H)-naftalenol, 1,3,4,5,6,8a-hexahidro-4,7-dimetil-1-(1-metiletil)-, (1S,4S,4aS,8aR)-(0. 68%), 2H-2,4a-ethanonaphthalen-8(5H)-one, hexahydro-2,5,5,5-trimethyl- (0.63%), β-vatirenone (0.56%), trans-calamenene (0.47%), γ-muurolene (0. 41%), γ-eudesmol (0.40%), nerolidol (0.35%), hedicariol (0.34%), cis-β-copaene (0.31%), cadin-3,5-diene (0. 31%), (+)-Isovalencenol (0.25%), α-agarofuran (0.23%), β-borbonene (0.20%), phytone (0.15%), pinocarvone (0.11%), (-)-myrtenol (0.09%)
Croton Thurifer (crotón)	Water drops, Jaen (17, 747094, 9371386, 681m)	0.03 (40)	-	-	-
Croton adipatus (crotón)	Water drops, Jaen (17, 747127, 9371220, 721m)	0.02 (43)	-	-	-
Aspidosperma poluneuron (acerillo)	Water drops, Jaen, (17, 747362, 9371130, 709m)	-	-	-	-
Luehea paniculata (falso roble)	Road to Loma Santa, Jaen (17, 741487, 9369943, 1060m)	-	-	-	-
Cybistax antisyphilitica (yangua)	Road to Loma Santa, Jaen (17, 741685, 9369430, 986m)	-	-	-	-

Zanthoxylum rigidum (teta de coche)	Shanango Sector, Jaen (17, 748800, 9377972, 717m)	-	-	-	-
Celtis loxensis (NI)	El Huito Sector, Jaen (17, 741955, 9370679, 922m)	-	-	-	-
Jatrofa humboldtiana (piñón)	Santa Cruz, Bellavista (17, 751871, 9374896, 518m)	-	-	-	-
Dictyoloma peruviana (barbasco)	Las Naranjas, Jaen (17, 738791, 9364496, 1344m)	-	-	-	-
Jacquinia mucronata (lishina)	Shanango Sector, Jaen (17, 749387, 9375996, 600m)	-	-	-	-
Cascabela thevetia (NI)	Linderos, Jaen (17, 748806, 9377964, 717m)	-	-	-	-
Adenaria floribunda (sinrugo)	Road to las Naranjas, Jaen (17, 739996, 9365124, 1287m)	-	-	-	-
Cordia lutea (overo)	Shanango Sector, Jaen (17, 749967, 9374955, 556m)	-	-	-	-
Croton xanthochylus (NI)	Water drops, Jaen (17, 745450, 9369595, 757m)	-	-	-	-
Trema micrantha (toropate)	Tabacal Sector, Jaén (17, 740256, 9365079, 1344m)	-	-	-	-
Muntingia calabura (cerezo)	Tabacal Sector, Jaen (17, 740316, 9365046, 1342m)	-	-	-	-
Cordia iguaguana (iguaguana)	Shanango Sector, Jaen (17, 748823, 9377993, 722m)	-	-	-	-
Guazuma Ulmifolia (NI)	Tabacal Sector, Jaen (17, 740331, 9365029, 1346m)	-	-	-	-
Handroanthus chrysanthus (huayacán)	Road to Loma Santa, Jaen (17, 747487, 9371508, 755m)	-	-	-	-
Capparis scabrida (zapote de perro)	Shanango Sector, Jaen (17, 749360, 9375982, 600m)	-	-	-	-
Handroanthus ochraceus (huayacán)	Road to Loma Santa, Jaen (17, 740242, 9370631, 1339m)	-	-	-	-

Note. Compound: identified by GC/MS compared to NIST library library 17. Relative abundance (%): relative amount of identified compounds as a function of the area of each peak in the total area of the chromatogram.
