# Supplementary material for: Discovering the Bioactive and Antibacterial Potential of Essential Oils from Aromatic Plants of Northeastern Peru
Source: Molecules. 2025 Oct 30;30(21):4236. doi: 10.3390/molecules30214236 (PMC12610044; doi:10.3390/molecules30214236)

Chromatographic profiles of essential oils

Esembeckia cornuta

t_R (min)	Compound	Relative abundance (%)	IR	IR ref
21.05	β-Thujene	0.06	934	966
24.13	Myrcene	0.19	990	991
25.61	α-Phellandrene	0.07	1018	1005
26.56	p-Cymene	0.06	1035	
27.23	Eucalyptol	0.11	1047	1032
30.16	Linalool	0.14	1101	1099
35.79	L-α-Terpineol	0.17	1210	
37.56	Neral	0.07	1246	
38.92	Citral	0.12	1273	1276
42.77	γ-Elemene	0.57	1354	1434
43.41	Cadina-3,5-diene	0.81	1367	1458
45.02	Copaene	3.94	1402	1376
45.38	(-)-cis-β-Elemene	6.58	1410	1391
45.56	β-Bourbonene	2.36	1414	1384
46.64	(-)-α-Gurjunene	0.29	1439	
47.10	β-Copaene	1.18	1450	1421
47.34	Caryophyllene	16.79	1455	1419
47.63	cis-β-Copaene	1.21	1462	1432
48.14	γ-Gurjunene	0.33	1473	1473
48.30	γ-Muurolene	0.73	1477	1477
48.92	1,5,9,9-Tetramethyl-1,4,7-cycloundecatriene	2.95	1491	1579
50.28	α-Amorphene	1.08	1523	1538
50.54	(-)-7-Epi-α-Selinene	2.43	1529	
50.82	Oxo-Tremorine	0.64	1536	
51.07	δ-Amorphene	14.58	1542	1524
51.23	(3S,3aR,3bR,4S,7R,7aR)-4-Isopropyl-3,7-dimethyloctahydro-1H-cyclopenta[1,3]cyclopropa[1,2]benzen-3-ol	0.66	1546	1515
51.36	trans-Calamenene	0.31	1549	1529
51.42	Liguloxide	1.16	1551	
52.17	Peruviol	6.23	1569	1564
52.31	β-Calacorene	0.45	1572	1563
52.34	Hedicariol	0.96	1573	
54.05	(1aR,4aR,7S,7aR,7bR)-1,1,7-Trimethyl-4-methylenedecahydro-1H-cyclopropa[e]azulen-7-ol	6.13	1615	1576
54.47	Caryophyllene oxide	2.40	1625	1581
54.91	Apiol	2.19	1637	1682
55.35	1H-Cycloprop[e]azulen-4-ol, decahydro-1,1,4,7-tetramethyl-, [1aR-(1aα,4β,4aβ,7α,7aβ,7bα)]-	0.72	1648	
55.92	τ-Cadinol	1.06	1662	1640
56.10	(±)-Cadinene	0.69	1667	1440
56.76	Humulane-1,6-dien-3-ol	5.50	1683	1619
56.94	α-Cadinol	1.13	1688	1653
57.06	5-Azulenemethanol, 1,2,3,4,5,6,7,8-octahydro-α,α,3,8-tetramethyl-, acetate	0.67	1691	1727

57.26	α-epi-7-epi-5-Eudesmol	2.03	1696	1598
57.87	1,4-Diisopropylbenzene	0.73	1712	1168
62.57	2-tert-Butylquinoline	1.01	1839	1552
63.19	Dehydrofukinone	0.35	1856	1817
65.71	Homosalate	0.19	1923	1904
65.86	Methyl palmitate	0.13	1927	1926
67.20	Palmitic Acid	0.56	1963	1968
68.49	2-Propenoic acid, 3-[4-[(3-methyl-1-butenyl)oxy]phenyl]-, methyl ester	1.95	1998	1798
74.44	Phytol	3.54	2158	2114
77.22	Hexadeca-2,6,10,14-tetraen-1-ol, 3,7,11,16-tetramethyl-	1.80	2233	
	Total	100.00		

Note. t_R (min): retention time. Compound: identified by GC/MS compared to NIST library libray 17. Relative abundance (%): relative amounts of identified compounds as a function of the area of each peak in the total area of the chromatogram. IR: relative retention indices calculated against n-alkanes. IR ref: retention index from literature.

Chromatographic profile on capillary column DB-5MS UI

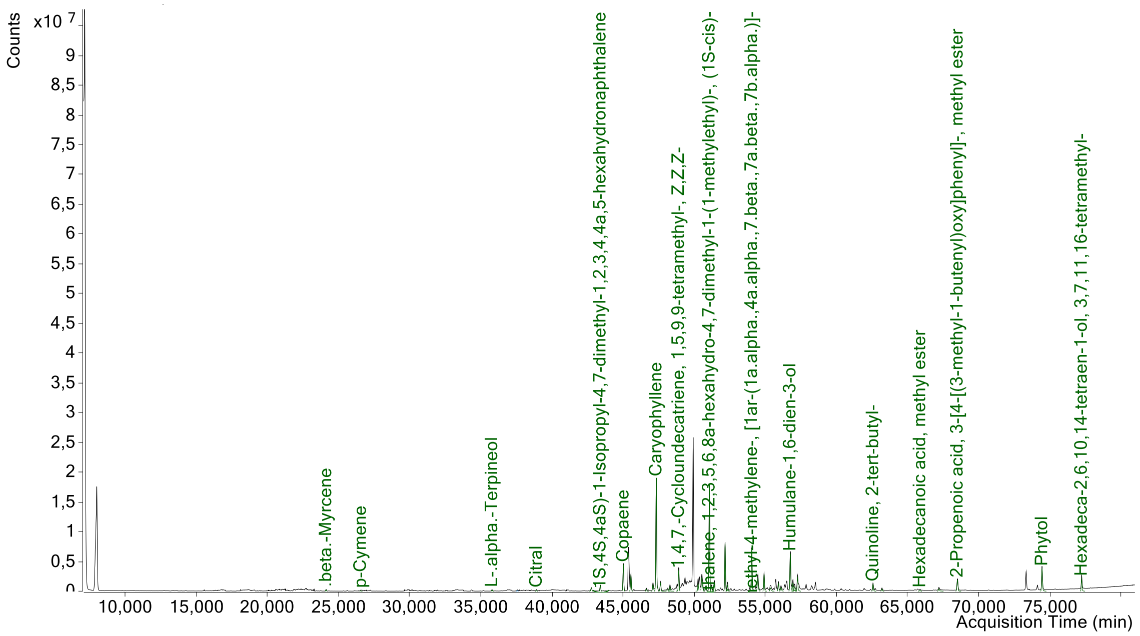

Magnolia Jaenensis

t_R (min)	Compound	Relative abundance (%)	IR	IR ref
8.01	2-Methyl-1-pentene	16.70	680	580
21.70	Cyclofenchene	0.11	946	
24.33	β-Pinene	0.25	994	
27.22	Eucalyptol	0.14	1048	1032
30.16	Linalool	0.12	1102	1099
35.79	L-α-Terpineol	0.11	1210	1190
37.56	Neral	0.28	1246	1240
38.62	trans-2-Decenal	0.02	1267	1263
38.91	Citral	0.46	1273	1270
39.85	Thymol	0.08	1292	1291
40.27	Isobornyl acetate	0.03	1301	
42.77	(+)-γ-Elemene	0.11	1354	1434
43.40	Cadina-3,5-diene	0.16	1368	1458
44.73	cis-β-Copaene	0.05	1396	1421
45.40	(-)-cis-β-Elemene	23.59	1411	1391
47.34	Caryophyllene	15.26	1455	1419
47.63	Bicyclosesquiphellandrene	0.58	1462	1489
48.14	Oxo-Tremorine	0.21	1474	1492
48.91	1,5,9,9-Tetramethyl-1,4,7-cycloundecatriene	2.64	1491	1579
49.35	γ-Muurolene	2.24	1501	1477
49.92	β-Copaene	0.74	1515	1432
50.28	α-Muurolene	1.09	1524	1499
50.38	β-Selinene	1.88	1526	1486
50.59	γ-Selinene	2.43	1531	1479
50.86	Myristicin	0.29	1538	1519
51.07	δ-Amorphene	10.15	1543	1524
51.22	(3S,3aR,3bR,4S,7R,7aR)-4-Isopropyl-3,7-dimethyloctahydro-1H-cyclopenta[1,3]cyclopropa[1,2]benzen-3-ol	0.19	1546	1515
51.55	trans-α-Bisabolene	0.24	1554	1512
51.69	(-)-7-Epi-α-Selinene	0.06	1558	1517
51.84	Cubebene	0.07	1561	1532
52.16	trans-Nerolidol	1.21	1569	1564
52.31	β-Calacorene	0.17	1573	1563
54.04	(1aR,4aR,7S,7aR,7bR)-1,1,7-Trimethyl-4-methylenedecahydro-1H-cyclopropa[e]azulen-7-ol	0.82	1615	1576
54.47	Caryophyllene oxide	1.07	1626	1581
54.92	Apiol	14.08	1637	1682
55.56	Humulene epoxide II	0.13	1654	1606
55.71	Selin-6-en-4α-ol	0.27	1657	1636
56.51	τ-Cadinol	0.85	1678	1640
56.93	α-Cadinol	0.29	1688	
57.37	Neointermedeol	0.61	1699	1660
58.13	Pentadecanal	0.08	1720	1715
59.05	6-Isopropenyl-4,8a-dimethyl-1,2,3,5,6,7,8,8a-octahydronaphthalen-2-ol	0.14	1745	1690

**Total**	100.00

Note. t_R (min): retention time. Compound: identified by GC/MS compared to NIST library libray 17. Relative abundance (%): relative amounts of identified compounds as a function of the area of each peak in the total area of the chromatogram. IR: relative retention indices calculated against n-alkanes. IR ref: retention index from literature.

Chromatographic profile on capillary column DB-5MS UI

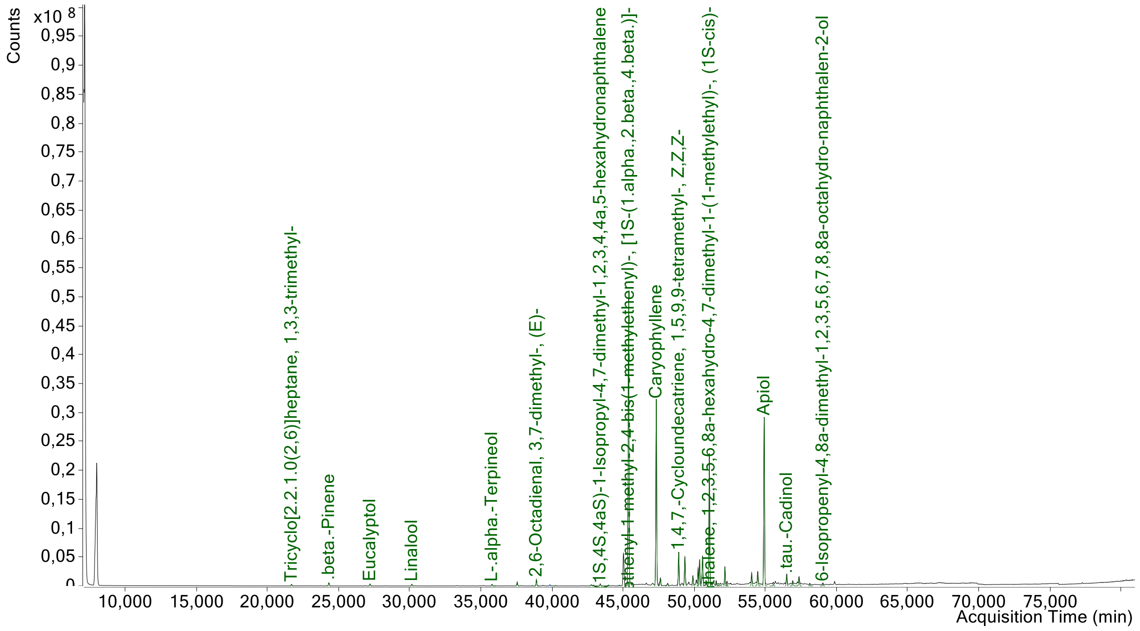

Magnolia manguillo

t_R (min)	Compound	Relative abundance (%)	IR	IR ref
16.78	cis-3-Hexen-1-ol	0.12	853	857
21.70	α-Thujene	0.06	946	929
23.77	β-Thujene	0.19	984	
24.12	Myrcene	0.05	991	991
24.33	β-Terpinene	0.41	994	1028
26.56	p-Cymene	0.16	1035	
26.87	1,4-Dimethyl-4-vinylcyclohexene	0.15	1041	
26.87	D-Limonene	0.25	1041	1018
30.16	Linalool	0.13	1102	
30.26	Perillene	0.07	1104	1101
33.37	Bicyclo[3.1.0]hexan-3-ol, 4-methylene-1-(1-methylethyl)-, [1S-(1α,3α,5α)]- (9CI)	0.23	1163	1143
35.18	4-Terpineol, (±)-	0.54	1198	1177
35.79	α-Terpineol	0.07	1210	1189
35.98	Myrtenol, (-)-	0.11	1214	1213
36.15	Myrtenal	0.19	1217	1193
37.56	Neral	0.05	1246	1240
38.92	Citral	0.09	1273	1270
43.40	α-Cubebene	1.21	1368	1351
45.02	Copaene	5.89	1403	1376
45.55	β-Bourbonene	0.73	1415	1384
46.63	(-)-α-Gurjunene	0.15	1439	
46.64	Cedrene-V6	0.21	1439	1440
47.33	Caryophyllene	11.59	1455	1419
47.63	cis-Muurola-4(15),5-diene	0.51	1462	
47.91	(-)-Guaia-6,9-diene	0.63	1468	
48.91	1,5,9,9-Tetramethyl-1,4,7-cycloundecatriene	4.81	1491	1579
49.35	γ-Muurolene	2.31	1501	1477
49.92	cis-β-Copaene	8.59	1515	1432
50.27	α-Muurolene	0.80	1523	1499
50.59	2-Isopropenyl-4a,8-dimethyl-1,2,3,4,4a,5,6,7-octahydronaphthalene	0.69	1531	1492
51.06	δ-Amorphene	4.44	1543	1524
51.22	(3S,3aR,3bR,4S,7R,7aR)-4-Isopropyl-3,7-dimethyloctahydro-1H-cyclopenta[1,3]cyclopropa[1,2]benzen-3-ol	0.72	1546	1515
51.36	Calamenene	0.89	1550	1523
52.17	trans-Nerolidol	6.46	1569	1564
52.31	β-Calacorene	0.38	1573	1563
52.55	Selina-3,7(11)-diene	0.31	1578	1542
53.08	Caryophyllene oxide	0.65	1591	1581
53.81	1,5-Epoxy-4(14)-salvilane	1.34	1609	1573

54.05	(1aR,4aR,7S,7aR,7bR)-1,1,7-Trimethyl-4-methylenedecahydro-1H-cyclopropa[e]azulen-7-ol	15.00	1615	1576
54.80	Salvil-4(14)-en-1-one	0.85	1634	1595
54.91	Apiol	0.49	1637	1682
55.39	(1R,7S,E)-7-Isopropyl-4,10-dimethylcyclodec-5-enol	3.03	1649	
55.56	Humulene epoxide II	2.19	1654	1606
55.79	Isospatulenol	3.89	1659	1638
56.08	(-)-Spathulenol	1.28	1667	1577
56.35	τ-Cadinol	2.38	1674	1640
56.94	α-Cadinol	4.94	1689	1653
58.30	Farnesol isomer A	1.17	1724	1710
58.52	7R,8R-8-Hydroxy-4-isopropylidene-7-methylbicyclo[5.3.1]undec-1-ene	2.76	1730	1754
59.05	3-(2-Isopropyl-5-methylphenyl)-2-methylpropanoic acid	0.94	1745	1745
59.87	6-Isopropenyl-4,8a-dimethyl-1,2,3,5,6,7,8,8a-octahydronaphthalen-2-ol	1.40	1767	1690
60.63	((4aS,8S,8aR)-8-Isopropyl-5-methyl-3,4,4a,7,8,8a-hexahydronaphthalen-2-yl)methanol	0.93	1787	
60.63	Ylangenol	0.82	1787	1666
62.00	((8R,8aS)-8-Isopropyl-5-methyl-3,4,6,7,8,8a-hexahydronaphthalen-2-yl)methanol	0.38	1824	1803
67.19	Palmitic acid	0.40	1964	1968
73.92	Phytol	0.97	2145	2114
	**Total**	100.00		

Note. t_R (min): retention time. Compound: identified by GC/MS compared to NIST library libray 17. Relative abundance (%): relative amounts of identified compounds as a function of the area of each peak in the total area of the chromatogram. IR: relative retention indices calculated against n-alkanes. IR ref: retention index from literature.

Chromatographic profile on capillary column DB-5MS UI

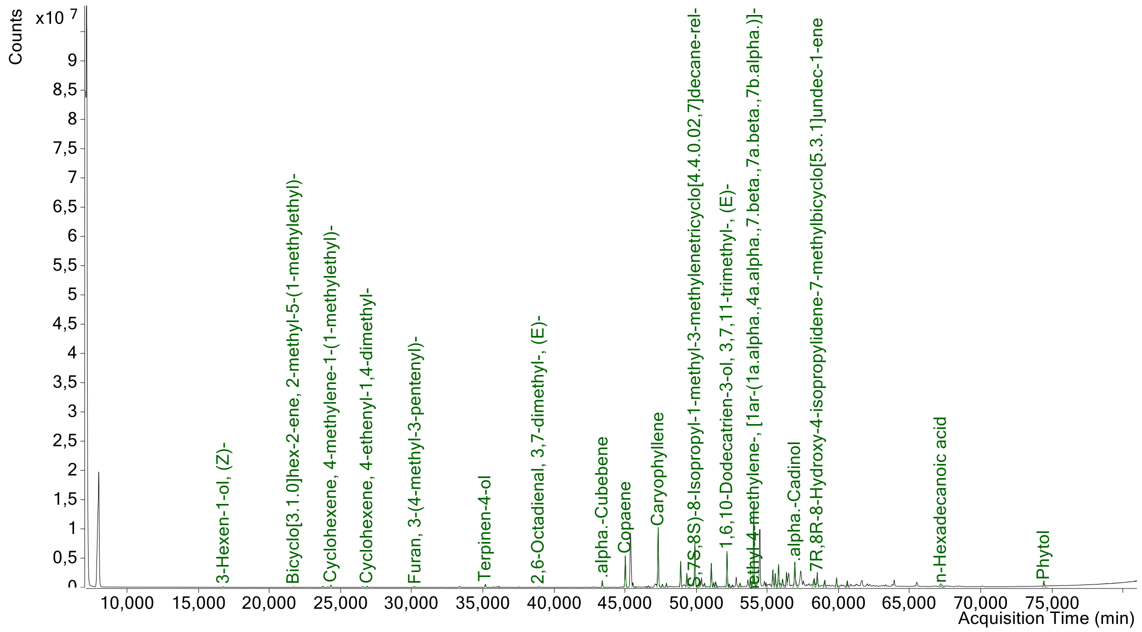

Piper aduncum

t_R (min)	Compound	Relative abundance (%)	IR	IR ref
45.02	Copaene	0.74	1403	1376
45.38	(-)-cis-β-Elemene	2.30	1411	1391
47.33	Caryophyllene	11.10	1455	1419
48.91	1,4,7-Cycloundecatriene, 1,5,9,9-tetramethyl-, Z,Z,Z-	2.47	1491	1579
49.30	Pentadecane	5.86	1500	1500
49.92	β-Copaene	3.12	1515	1432
50.55	α-Elemene	1.96	1530	1462
50.86	Myristicin	38.26	1538	1519
51.07	(+)-δ-Cadinene	2.01	1543	1524
52.17	trans-Nerolidol	0.73	1569	1564
53.92	τ-Cadinol	1.68	1612	1640
54.04	1H-Cycloprop[e]azulen-7-ol, decahydro-1,1,7-trimethyl-4-methylene-, [1aR-(1aα,4aα,7β,7aβ,7bα)]-	10.04	1615	1576
54.47	Isoaromadendrene epoxide	8.40	1626	
55.57	Humulene epoxide II	1.62	1654	1606
55.72	(3R,3aR,3bR,4S,7R,7aR)-4-Isopropyl-3,7-dimethyloctahydro-1H-cyclopenta[1,3]cyclopropa[1,2]benzen-3-ol	6.63	1658	1493
57.00	Apiol	3.08	1690	1682
	**Total**	100.00		

Note. t_R (min): retention time. Compound: identified by GC/MS compared to NIST library libray 17. Relative abundance (%): relative amounts of identified compounds as a function of the area of each peak in the total area of the chromatogram. IR: relative retention indices calculated against n-alkanes. IR ref: retention index from literature.

Chromatographic profile on capillary column DB-5MS UI

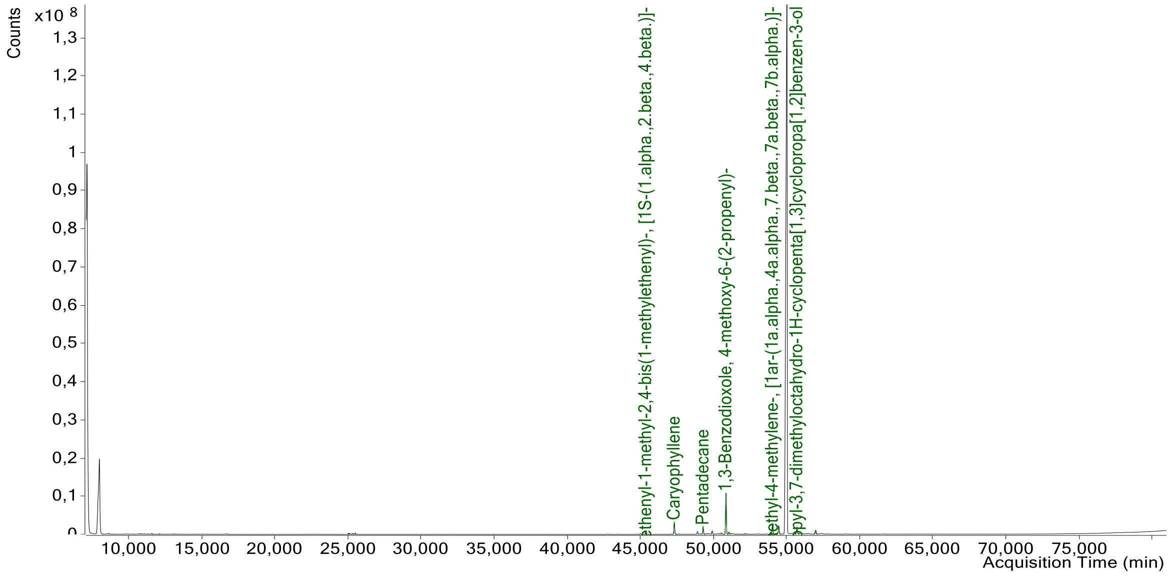

Piper amalogo

t_R (min)	Compound	Relative abundance (%)	IR	IR ref
21.05	α-Thujene	0.08	934	929
21.71	Cyclofenchene	6.02	946	890
22.80	Camphene	0.08	966	
23.77	Sabinene	0.15	984	
24.13	β-Myrcene	0.29	991	991
24.33	β-Pinene	9.96	995	943
26.16	α-Terpinene	0.08	1028	1017
26.56	p-Cymene	0.28	1035	1025
26.87	D-Limonene	0.71	1041	1018
27.23	Eucalyptol	4.50	1048	1032
28.33	γ-Terpinene	0.15	1068	1060
29.91	α-Terpinolene	0.08	1097	1088
33.37	(+)-Sabinol	0.17	1163	1143
34.96	(+)-Borneol	0.16	1194	
35.18	(-)-Terpinen-4-ol	0.40	1198	1182
35.79	L-α-Terpineol	1.73	1210	1190
35.99	(-)-Myrtenol	0.10	1214	1213
36.15	α-Thujenal	0.05	1218	1190
37.57	Neral	0.04	1246	1240
38.92	Citral	0.06	1273	1276
40.68	(1S,3S,5S)-1-Isopropyl-4-methylenebicyclo[3.1.0]hexan-3-yl acetate	0.02	1310	1297
41.97	Myrtenyl acetate	0.03	1337	1327
42.14	Theaspirane	0.06	1341	1302
42.79	δ-Elemene	0.12	1355	1338
42.99	α-Terpinyl acetate	0.22	1359	1350
43.41	(-)-α-Cubebene	1.73	1368	
44.73	(+)-Ylangene	0.55	1396	1372
45.02	Copaene	3.16	1403	1376
45.38	β-Elemene	0.78	1411	
45.56	(-)-β-Bourbonene	0.24	1415	1384
46.63	(-)-α-Gurjunene	0.84	1439	1409
47.10	β-Copaene	1.68	1450	1421
47.33	Caryophyllene	3.23	1455	1419
47.64	β-Copaene (Note: This is a duplicate entry)	4.21	1462	1432
47.91	(+)-Calarene	0.18	1468	1432
48.14	Naphthalene, 1,2,3,5,6,7,8,8a-octahydro-1,8a-dimethyl-7-(1-methylethenyl)-, [1R-(1α,7β,8aα)]-	1.70	1474	1492
48.46	Cadina-3,5-diene	0.19	1481	1458
48.92	1,4,7-Cycloundecatriene, 1,5,9,9-tetramethyl-, Z,Z,Z-	10.50	1491	1579
49.07	1-epi-Bicyclosesquiphellandrene	0.52	1495	1489
49.36	γ-Muurolene	8.26	1501	1477
50.28	α-Muurolene	0.83	1524	1499
50.39	(3R,3aR,3bR,4S,7R,7aR)-4-Isopropyl-3,7-dimethyloctahydro-1H-cyclopenta[1,3]cyclopropa[1,2]benzen-3-ol	2.34	1526	
50.86	Myristicin	0.15	1538	1519

51.07	(+)-δ-Cadinene	13.63	1543	1524
51.36	(+)-trans-Calamenene	1.31	1550	1529
51.84	Naphthalene, 1,2,3,4,4a,7-hexahydro-1,6-dimethyl-4-(1-methylethyl)-	0.53	1561	1533
51.99	α-Amorphene	0.37	1565	1538
52.17	Peruviol	0.07	1569	1564
52.32	β-Calacorene	0.71	1573	1563
54.05	1H-Cycloprop[e]azulen-7-ol, decahydro-1,1,7-trimethyl-4-methylene-, [1aR-(1aα,4aα,7β,7aβ,7bα)]-	3.73	1615	1576
54.92	Apiol	7.21	1637	1682
55.56	Humulene epoxide II	1.47	1654	1606
55.91	4a(2H)-Naphthalenol, 1,3,4,5,6,8a-hexahydro-4,7-dimethyl-1-(1-methylethyl)-, (1S,4S,4aS,8aR)-	0.69	1663	1614
56.35	τ-Cadinol	0.41	1674	1640
56.52	3,8-Dimethyl-5-α-hydroxy-δ^9-octahydroazulene acetate	0.93	1678	
56.94	α-Cadinol	0.76	1689	
57.27	α-epi-7-epi-5-Eudesmol	1.40	1697	1598
58.52	7R,8R-8-Hydroxy-4-isopropylidene-7-methylbicyclo[5.3.1]undec-1-ene	0.16	1730	
	**Total**	100.00		

Note. t_R (min): retention time. Compound: identified by GC/MS compared to NIST library libray 17. Relative abundance (%): relative amounts of identified compounds as a function of the area of each peak in the total area of the chromatogram. IR: relative retention indices calculated against n-alkanes. IR ref: retention index from literature.

Chromatographic profile on capillary column DB-5MS UI

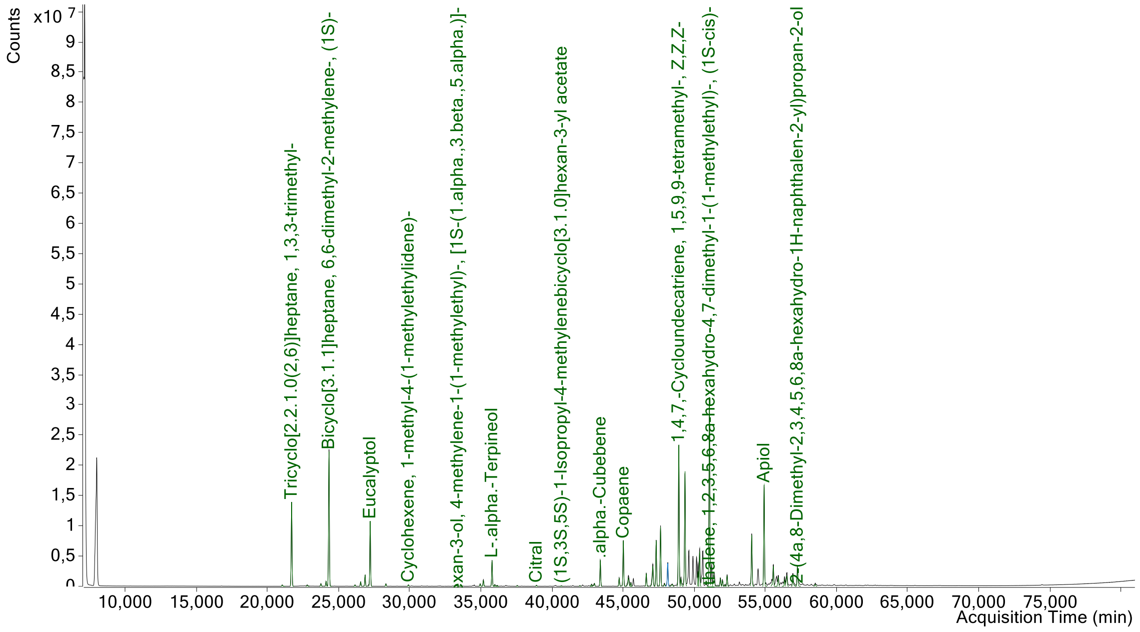

Piper glabribaccum

t_R (min)	Compound	Relative abundance (%)	IR	IR ref
23.20	Benzaldehyde	0.01	974	962
23.74	6-Methyl-5-hepten-2-one	0.49	984	986
30.42	6-Methyl-3,5-heptadien-2-one	0.01	1107	
38.92	Citral	0.09	1273	1276
42.76	γ-Elemene	0.13	1354	1434
43.41	(-)-α-Cubebene	14.30	1368	1351
44.60	1,2,9,10-Tetradehydroaristolane	0.97	1394	1435
44.78	Isoledene	0.09	1397	1375
45.02	Copaene	5.25	1403	1376
45.38	(-)-cis-β-Elemene	9.63	1411	1391
46.64	(-)-α-Gurjunene	5.22	1439	1409
46.99	Disoxilonene	0.11	1447	1440
47.14	(1S,2E,6E,10R)-3,7,11,11-Tetramethylbicyclo[8.1.0]undeca-2,6-diene	0.51	1451	1495
47.33	Bicyclo[5.2.0]nonane, 2-methylene-4,8,8-trimethyl-4-vinyl-	4.93	1455	1407
47.57	Z-β-Guaiene	0.85	1461	1439
48.14	Alloaromadendrene	2.85	1474	
48.92	1,4,7-Cycloundecatriene, 1,5,9,9-tetramethyl-, Z,Z,Z-	3.54	1491	1579
49.14	γ-Gurjunene	0.80	1496	1473
49.37	γ-Selinene	1.60	1502	
50.27	4a,5-Dimethyl-3-(prop-1-en-2-yl)-1,2,3,4,4a,5,6,7-octahydronaphthalene	2.12	1523	1492
50.60	α-Selinene	4.51	1531	1474
51.07	Cadina-1(10),4-diene	4.30	1543	1524
51.23	(3R,3aR,3bR,4S,7R,7aR)-4-Isopropyl-3,7-dimethyloctahydro-1H-cyclopenta[1,3]cyclopropa[1,2]benzen-3-ol	2.17	1546	1493
51.36	trans-Calamenene	1.46	1550	1529
51.84	Cubebene	0.18	1561	1532
52.00	α-Amorphene	0.16	1565	1538
52.19	trans-Nerolidol	11.46	1570	1564
52.32	β-Calacorene	1.10	1573	1563
53.08	Isoaromadendrene epoxide	0.18	1591	
53.46	1H-Cycloprop[e]azulen-4-ol, decahydro-1,1,4,7-tetramethyl-, [1aR-(1aα,4β,4aβ,7α,7aβ,7bα)]-	0.39	1600	1591
53.93	τ-Cadinol	1.35	1612	1640
54.05	1H-Cycloprop[e]azulen-7-ol, decahydro-1,1,7-trimethyl-4-methylene-, [1aR-(1aα,4aα,7β,7aβ,7bα)]-	3.62	1615	1576
54.50	Guaiol	8.19	1627	1596
55.22	α-Corocalene	0.33	1645	1623
55.35	(-)-Globulol	1.06	1648	
55.57	Humulene epoxide II	1.14	1654	1606
57.37	Eudesm-7(11)-en-4-ol	1.67	1700	1692
57.63	Cadalene	2.08	1706	1674
59.05	7R,8R-8-Hydroxy-4-isopropylidene-7-methylbicyclo[5.3.1]undec-1-ene	0.59	1745	1754

59.61	6-Isopropenyl-4,8a-dimethyl-1,2,3,5,6,7,8,8a-octahydronaphthalen-2-ol	0.22	1760	1690
62.72	Hexahydrofarnesyl acetone	0.06	1843	1844
73.40	1-Octadecanol	0.09	2131	2082
74.45	Phytol	0.19	2159	2114
	**Total**	100.00		

Note. t_R (min): retention time. Compound: identified by GC/MS compared to NIST library libray 17. Relative abundance (%): relative amounts of identified compounds as a function of the area of each peak in the total area of the chromatogram. IR: relative retention indices calculated against n-alkanes. IR ref: retention index from literatura.

Chromatographic profile on capillary column DB-5MS UI

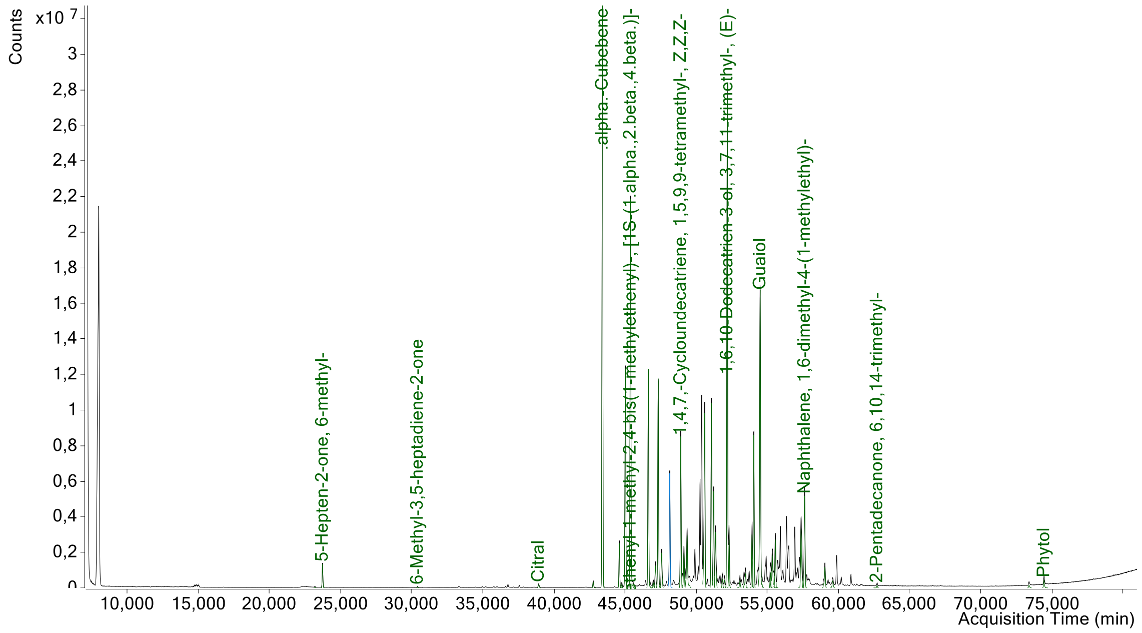

Tesaria integrifolia

t_R (min)	Compound	Relative abundance (%)	IR	IR ref
33.38	(+)-Sabinol	0.91	1163	1143
34.43	Pinocarvone	0.11	1184	1164
35.99	(-)-Myrtenol	0.09	1214	1213
43.40	Cadina-3,5-diene	0.31	1368	1458
45.02	Copaene	1.53	1403	1376
45.38	(-)-cis-β-Elemene	1.81	1411	1391
45.55	β-Bourbonene	0.20	1415	1384
47.33	Caryophyllene	9.00	1455	1419
47.63	cis-β-Copaene	0.31	1462	1432
48.81	Phenol, 2,4-bis(1,1-dimethylethyl)-6-methyl-	10.01	1489	1676
48.91	1,5,9,9-Tetramethyl-1,4,7-cycloundecatriene	1.22	1491	1579
49.35	γ-Muurolene	0.41	1501	
50.38	(3R,3aR,3bR,4S,7R,7aR)-4-Isopropyl-3,7-dimethyloctahydro-1H-cyclopenta[1,3]cyclopropa[1,2]benzen-3-ol	2.03	1526	
51.06	δ-Amorphene	2.11	1543	1524
51.35	trans-Calamenene	0.47	1550	1529
51.42	Dihydroagarofuran	8.08	1551	1496
52.17	Nerolidol	0.35	1569	
52.35	Hedicariol	0.34	1573	
53.13	α-Agarofuran	0.23	1592	1550
54.04	(1aR,4aR,7S,7aR,7bR)-1,1,7-Trimethyl-4-methylenedecahydro-1H-cyclopropa[e]azulen-7-ol	1.67	1615	1576
54.34	4,4a,5,6,7,8-Hexahydro-4a,8-dimethylnaphthalen-2(3H)-one	6.71	1623	1418
54.47	Caryophyllene oxide	2.09	1626	1581
54.91	Apiol	1.01	1637	1682
55.05	Oplopenone	0.97	1641	1606
55.91	4a(2H)-Naphthalenol, 1,3,4,5,6,8a-hexahydro-4,7-dimethyl-1-(1-methylethyl)-, (1S,4S,4aS,8aR)-	0.68	1662	1614
56.04	γ-Eudesmol	0.40	1666	1631
56.34	Agarospirol	2.14	1673	1645
57.07	3,8-Dimethyl-5-α-hydroxy-δ^9-octahydroazulene acetate	8.74	1692	1727
57.28	2-Naphthalenemethanol, decahydro-α,α,4a-trimethyl-8-methylene-, [2R-(2α,4aα,8aβ)]-	18.28	1697	1649
57.87	6,7-Dimethyl-1,2,3,5,8,8a-hexahydronaphthalene	8.63	1713	1273
58.15	β-Vetivone	0.56	1721	1540
59.25	2H-2,4a-Ethanonaphthalen-8(5H)-one, hexahydro-2,5,5-trimethyl-	0.63	1750	1512
59.56	Naphthalene, 1,2,3,4-tetrahydro-1,1,2,4,4,7-hexamethyl-	0.75	1758	1610
61.55	(+)-Isovalencenol	0.25	1812	
62.71	Phytone	0.15	1843	1844

63.19	Dehydrofukinone	6.82	1856	1817
	**Total**	100.00		

Note. t_R (min): retention time. Compound: identified by GC/MS compared to NIST library libray 17. Relative abundance (%): relative amounts of identified compounds as a function of the area of each peak in the total area of the chromatogram. IR: relative retention indices calculated against n-alkanes. IR ref: retention index from literature.

Chromatographic profile on capillary column DB-5MS UI

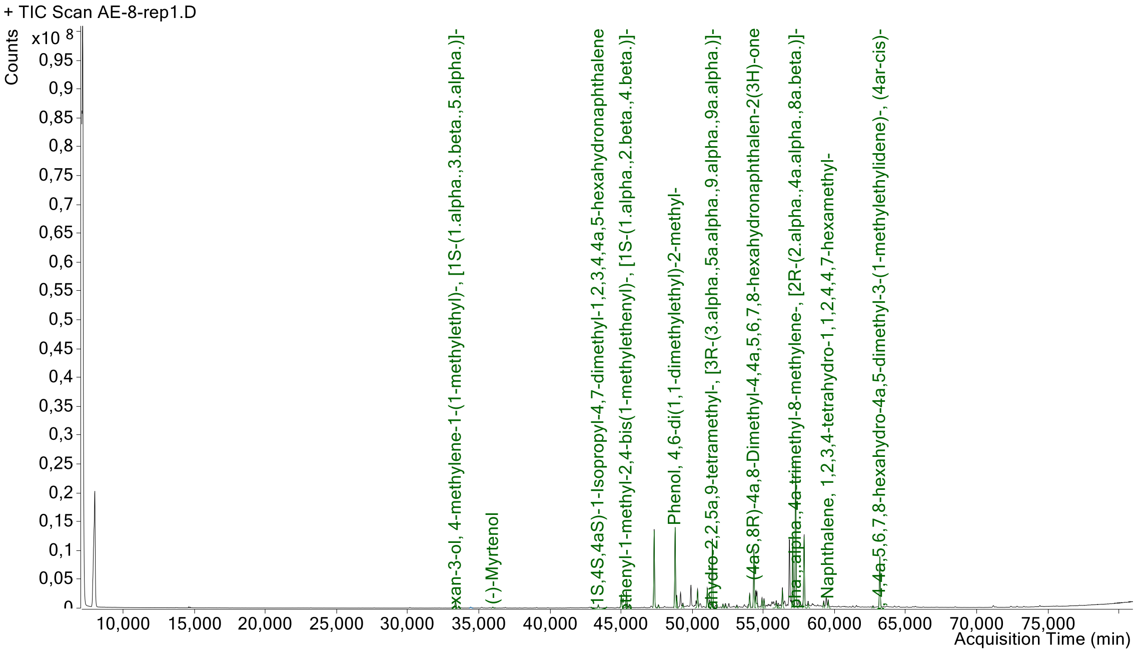

Zanthoxylum fagara

t_R (min)	Compound	Relative abundance (%)	IR	IR ref
24.3	Sabinene	0.07	994	974
25.6	α-Phellandrene	0.07	1018	1005
26.6	p-Cymene	0.05	1035	
26.9	D-Limonene	0.08	1041	1018
42.8	δ-Elemene	0.99	1355	1338
43.4	(-)-α-Cubebene	0.74	1368	
45.0	Copaene	3.26	1403	
45.4	(-)-cis-β-Elemene	9.08	1411	1391
45.6	β-Bourbonene	0.16	1415	1384
46.6	(-)-α-Gurjunene	0.09	1439	1409
47.1	γ-Elemene	0.96	1451	
47.4	Caryophyllene	40.00	1456	1419
47.6	cis-β-Copaene	1.07	1462	1432
48.9	1,5,9,9-Tetramethyl-1,4,7-cycloundecatriene	5.86	1491	1579
49.4	γ-Muurolene	1.20	1501	1477
50.3	α-Muurolene	2.17	1524	1499
50.5	(1S,2E,6E,10R)-3,7,11,11-Tetramethylbicyclo[8.1.0]undeca-2,6-diene	2.05	1530	
51.1	δ-Amorphene	3.77	1543	1524
51.2	(3S,3aR,3bR,4S,7R,7aR)-4-Isopropyl-3,7-dimethyloctahydro-1H-cyclopenta[1,3]cyclopropa[1,2]benzen-3-ol	0.49	1546	1515
51.4	trans-Calamenene	0.17	1550	1529
52.2	Nerolidol	0.42	1569	1564
52.4	Hedicariol	10.86	1574	
53.9	τ-Cadinol	0.18	1612	1640
54.0	(1aR,4aR,7S,7aR,7bR)-1,1,7-Trimethyl-4-methylenedecahydro-1H-cyclopropa[e]azulen-7-ol	1.63	1615	1576
54.5	Guaiol	4.48	1626	1596
55.5	(1S,3aS,4S,5S,7aR,8R)-5-Isopropyl-1,7a-dimethyloctahydro-1H-1,4-methanoinden-8-ol	0.20	1651	1534
55.9	γ-Gurjunene	0.72	1662	1473
56.1	γ-Eudesmol	1.84	1668	1631
56.4	5-Azulenemethanol, 1,2,3,4,5,6,7,8-octahydro-α,α,3,8-tetramethyl-, acetate	0.23	1674	
56.9	α-Cadinol	1.00	1689	1653
57.3	α-epi-7-epi-5-Eudesmol	3.10	1697	1598
57.4	Bulnesol	1.73	1699	1667
57.8	Ledol	0.87	1710	1565
60.3	β-Selinene	0.41	1777	1486
	**Total**	100.00		

Note. t_R (min): retention time. Compound: identified by GC/MS compared to NIST library libray 17. Relative abundance (%): relative amounts of identified compounds as a function of the area of each peak in the total area of the chromatogram. IR: relative retention indices calculated against n-alkanes. IR ref: retention index from literature.

Chromatographic profile on capillary column DB-5MS UI

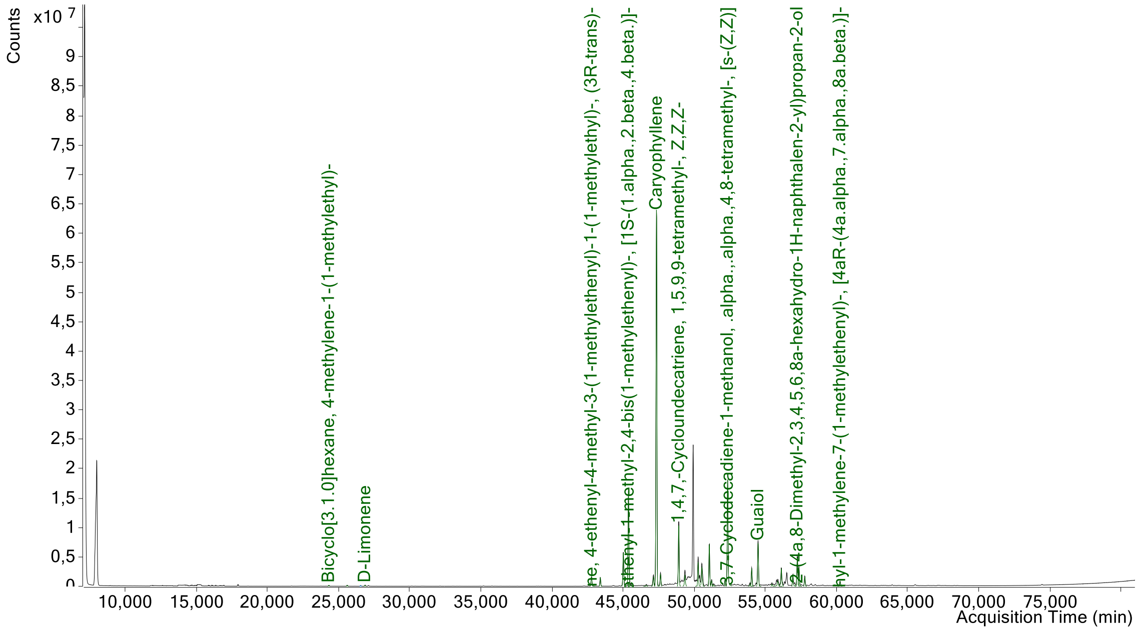

Supplement: Supplementary file 1 [file molecules-30-04236-s001.zip › Table S2. Chromatographic profiles (and chromatogram) of essential oils_Corrected.pdf]
